# Parcellation of the primary cerebral cortices based on local connectivity profiles

**DOI:** 10.3389/fnana.2015.00050

**Published:** 2015-04-27

**Authors:** Qiaojun Li, Ming Song, Lingzhong Fan, Yong Liu, Tianzi Jiang

**Affiliations:** ^1^Brainnetome Center, Institute of Automation, Chinese Academy of SciencesBeijing, China; ^2^National Laboratory of Pattern Recognition, Institute of Automation, Chinese Academy of SciencesBeijing, China; ^3^CAS Center for Excellence in Brain Science, Institute of Automation, Chinese Academy of SciencesBeijing, China; ^4^The Queensland Brain Institute, University of Queensland, BrisbaneQLD, Australia

**Keywords:** primary cerebral cortices, connectivity-based parcellation, local connectivity profiles, diffusion tensor imaging, tractography

## Abstract

Connectivity-based parcellation using diffusion MRI has been extensively used to parcellate subcortical areas and the association cortex. Connectivity profiles are vital for connectivity-based parcellation. Two categories of connectivity profiles are generally utilized, including global connectivity profiles, in which the connectivity information is from the seed to the whole brain, and long connectivity profiles, in which the connectivity information is from the seed to other brain regions after excluding the seed. However, whether global or long connectivity profiles should be applied in parcellating the primary cortex utilizing connectivity-based parcellation is unclear. Many sources of evidence have indicated that the primary cerebral cortices are composed of structurally and functionally distinct subregions. Because the primary cerebral cortices are rich in local anatomic hierarchical connections and possess high degree of local functional connectivity profiles, we proposed that local connectivity profiles, that is the connectivity information within a seed region of interest, might be used for parcellating the primary cerebral cortices. In this study, the global, long, and local connectivity profiles were separately used to parcellate the bilateral M1, A1, S1, and V1. We found that results using the three profiles were all quite consistent with reported cytoarchitectonic evidence. More importantly, the results using local connectivity profiles showed less inter-subject variability than the results using the other two, a finding which suggests that local connectivity profiles are superior to global and long connectivity profiles for parcellating the primary cerebral cortices. This also implies that, depending on the characteristics of specific areas of the cerebral cortex, different connectivity profiles may need to be adopted to parcellate different areas.

## Introduction

Exploring the structural and functional organization of the brain is one of the most prominent research endeavors in neuroscience. Mapping its subdivisions is a very important aspect of exploring the human brain (Cloutman and Lambon Ralph, 2012). The primary cerebral cortices, including the primary motor (M1), primary somatosensory (S1), primary auditory (A1), and primary visual (V1) cortices, are specialized processing centers for dealing with related primary information (O’Leary et al., 2007). Exploring the fine structure of the primary cerebral cortices has long been an important goal for researchers in the neural sciences. A large amount of work has been done using post-mortem histological tools (Geyer et al., 2000; Schönwiesner et al., 2002) or functional methods (Binkofski et al., 2002; Blankenburg et al., 2003; Formisano et al., 2003). For example, the M1 was identified as having two subregions using the quantitative cytoarchitectonic method (Geyer et al., 1996). The S1 was divided cytoarchitectonically into four areas, 3a, 3b, 1, and 2 (Schönwiesner et al., 2002), and the A1 was segregated into two areas (Galaburda and Sanides, 1980) or three areas (Morosan et al., 2001; Upadhyay et al., 2007). These studies provide evidence that the primary cerebral cortices are indeed composed of structurally and functionally distinct subregions.

Structural connectivity-based parcellation using diffusion MRI can non-invasively subdivide regions *in vivo* and has been extensively applied to parcellate the subcortical areas and association cortices (see Cloutman and Lambon Ralph, 2012, for a review). The results obtained from structural connectivity-based parcellation tend to resemble the results from functional and cytoarchitectonic studies (Knosche and Tittgemeyer, 2011; Cloutman and Lambon Ralph, 2012). This provides important evidence that structural connectivity-based parcellation is indeed capable of representing the fine substructure of cortical regions. In structural connectivity-based parcellation studies, the formation of connectivity profiles is crucial for the eventual parcellation. Two categories of connectivity profiles, that is, global connectivity profiles and long connectivity profiles, are generally utilized. Global connectivity profiles encompass connectivity information from the seed to the whole brain (Klein et al., 2007; Mars et al., 2011) and long connectivity profiles encompass connectivity information from the seed to the other brain regions after excluding the seed region (Anwander et al., 2007; Beckmann et al., 2009; Wang et al., 2012; Fan et al., 2014). Since the subcortical areas and association cortex receive widespread projections from the distributed brain system, global and long connectivity profiles can adequately represent their characteristics (Mesulam, 1990, 2008). However, the primary cerebral cortices are heavily myelinated (Glasser and Van Essen, 2011; Glasser et al., 2014), rich in local hierarchical connections (Felleman and Van Essen, 1991), and possess a high density of local functional connectivity profiles (Sepulcre et al., 2010). Therefore, using connectivity-based parcellation to form global or long connectivity profiles and then applying these to parcellate the primary cerebral cortices may not be ideal given the nature of the primary cortices. In contrast, parcellating the primary cerebral cortices using local connectivity profiles, that is, the connectivity information within the primary cerebral cortices, could be quite interesting.

To investigate this, we chose the M1, S1, A1, and V1 as regions of interest (ROIs) and compared them using the three kinds of connectivity profiles (global, long, and local). We first constructed a connectivity matrix for each of the three profiles and then clustered the profiles which subdivided the seed ROIs on two independent datasets. We also parcellated the four primary cerebral cortices based on resting-state functional magnetic resonance imaging (fMRI). The results for each seed ROI were compared with the cytoarchitectonic results from FZ Jülich’s SPM Anatomy toolbox (Eickhoff et al., 2005) and with the results from the resting-state fMRI. In addition we compared the results between the three kinds of connectivity profiles. Using this technique we were able to evaluate the usefulness of the different types of profiles for parcellating the primary cerebral cortices and, in particular, to test our hypothesis that local connectivity might be particularly useful in these regions.

## Materials and Methods

### Subjects and MRI Data Acquisition

Two independent datasets were used in this study. Dataset 1 included 20 healthy, right-handed subjects (10 males and 10 females, mean age: 18.5 years, range: 17–20 years). All participants provided written informed consent. None of the participants had ever suffered from any psychiatric or neurological disease, and none had any contraindications for MRI scanning. Diffusion-weighted images, T1-weighted images, and resting-state functional magnetic resonance imaging (fMRI) were acquired on a 3.0 T GE MR scanner (General Electric, Milwaukee, WI, USA). The diffusion-weighted images were acquired using spin-echo echo-planar imaging (TR = 8.5 s, T = minimum, 75 axial slices, resolution = 2 mm × 2 mm × 2 mm, FOV = 256 mm × 256 mm) in non-collinear 64 directions (*b* = 1000 s/mm^2^), along with one non-diffusion weighted volume (*b* = 0 s/mm^2^). The T1-weighted images were acquired using a Sag 3D BRAVO sequence (TR = 1.9 s, TE = 3 ms; FOV = 256 mm × 256 mm; in-plane resolution = 1 mm × 1 mm; slice thickness = 1 mm; 192 slices). The resting-state fMRI images were obtained using a gradient-echo single-shot echo-planar imaging sequence (GE-EPI) with the following imaging parameters: TR/TE = 2000/30 ms; FOV = 240 mm × 240 mm; matrix = 64 × 64; FA = 90°, slice thickness = 3.4 mm; 0.6 mm gap; 39 transversal slices; 255 volumes. All aspects of the study were approved by the Ethics Committee of Tianjin Medical University.

Dataset 2 was part of the Enhanced Nathan Kline Institute (NKI)/Rockland lifespan sample (Nooner et al., 2012)^1^. It included 20 healthy, right-handed subjects (10 males and 10 females, mean age: 22.8 years, range: 17–44 years). Diffusion-weighted data and high-resolution T1-weighted images were acquired using a 3.0 Tesla Siemens TrioTim scanner at NKI. The diffusion-weighted data were acquired using echo-planar imaging (TR = 2.4 s; TE = 85 ms; 64 axial slices; resolution = 2 mm × 2 mm × 2 mm; FOV = 212 mm × 212 mm) in 137 non-collinear directions (*b*-value = 1000 s/mm^2^), along with one non-diffusion weighted volume (*b* = 0 s/mm^2^). The high-resolution T1-weighted images were acquired by a brain volume sequence (TR = 1.9 s, TE = 2.52 ms; FOV = 250 mm × 250 mm; in-plane resolution = 1 mm × 1 mm; slice thickness = 1 mm; 176 slices).

### Preprocessing of DTI and fMRI Images

The diffusion and structural MR images were preprocessed using FMRIB’s Diffusion Toolbox (FSL 4.1^2^) and included the following steps: (1) correcting for eddy currents and head motion; (2) co-registering the skull-stripped T1-weighted image to the *b* = 0 images in native DTI space and then transforming to Montreal Neurological Institute (MNI) space; and (3) transforming the seed masks from MNI space to the native DTI space with nearest-neighbor interpolation using the inverted transformation parameters obtained above.

The resting-state fMRI images were preprocessed using the following steps: (1) discarding the first ten volumes; (2) correcting for slice timing and head motion; (3) intensity scaling of the fMRI images; (4) spatially smoothing and temporally band-pass filtering; and (5) removing nuisance signals, including the signal averaged from the whole-brain mask, the signal averaged from the white matter mask, and the signal averaged from the ventricular mask, six motion parameters, and their first derivatives.

### Definition of ROIs

There are many different definitions for the location of the primary cerebral cortices, and the number of subregions in the primary cerebral cortices has varied in previous studies. Because some researchers have suggested that architectonic and probability maps must currently be considered as state of the art for cortical parcellation (Wasserthal et al., 2014), in this study we used the ROIs obtained using FZ Jülich’s SPM toolbox^3^ (Eickhoff et al., 2005) as the seed masks and calculated the overlap between our parcellation results and the parcellation results this toolbox provided.

The detailed definitions of the M1, S1, A1, and V1 that we used were as follows. The M1 corresponded to BA 4 (Geyer et al., 1996) and two subregions were identified. We chose BA 3 as a seed region for S1, based on a recent study (Keysers et al., 2010), which described this region as being homogenous with the other sensory cortices. Also using other’s results (Schönwiesner et al., 2002) as guidance, we subdivided BA 3 into two subregions. A1 was chosen as Te1 (Morosan et al., 2001) and three subregions were identified. We defined BA 17 (Amunts et al., 2000) as V1. Based on the research by Boucard et al. (2009) and Yu et al. (2014), we identified the number of subregions of V1 as 2. Bilateral seed ROIs for these primary cerebral cortices were obtained using FZ Jülich’s toolbox. These seed ROIs were transformed into MNI space by an affine registration using FSL (Jenkinson and Smith, 2001).

### Parcellation of the Primary Cerebral Cortices based on Diffusion MRI

Voxelwise estimates of the fiber orientation distribution were calculated using Bedpostx within FSL. The probability distributions for two fiber directions at each voxel were calculated using a multiple fiber extension (Behrens et al., 2007) of a previously published diffusion modeling approach (Triarhou, 2007). Thus, the fiber tracts between each voxel in the seed region and every voxel of the whole brain were estimated. We sampled 5000 streamline fibers per voxel to estimate the connectivity probability. The connectivity profile of each voxel in the seed ROI was then used to construct the connectivity matrix (Johansen-Berg et al., 2004). If the number of voxels in the ROI mask is defined as M and the number of voxels in the brain mask is N, the method for calculating the global connectivity profiles included all the connectivity information from the ROI mask to the whole brain, resulting in an M × N connectivity matrix. Using the same designations, the method for calculating the long connectivity profiles included all the rest of the information from the whole brain after excluding the ROI information, resulting in an M × (N-M) connectivity matrix. Similarly, the method using local connectivity profiles included only the connectivity information within the ROI mask, resulting in an M × M connectivity matrix. In this way, three types of cross-correlation (CC) matrices were formed for each subject. Subsequently, spectral clustering was introduced to automatically cluster the CC matrices in order to obtain distinct subregions (Ng et al., 2002). Finally, all the subregions were transformed back into MNI space and a maximum probability map (MPM; Eickhoff et al., 2005) was obtained. This entire process was applied to all the selected ROIs.

### Parcellation of the Primary Cerebral Cortices using Resting-State fMRI

The M1, A1, S1, and V1 were parcellated using resting-state fMRI to test the viability of local connectivity profiles. In order to be able to compare these results with those based on connectivity-based parcellation using the local connectivity profiles, we again parcellated the M1, S1, and V1 into two subregions and the A1 into three subregions. The parcellation based on the resting-state fMRI data included the following steps: (1) calculating the Pearson correlation between voxels in the seed region; (2) constructing the correlation matrix; (3) clustering the correlation matrix; and (4) calculating the MPM of the parcellation results for each parcellated region.

### Calculation of Consistency and Stability

The consistency and stability of the results obtained from various subjects are often used to justify the acceptance of a parcellation (Kahnt et al., 2012; Li et al., 2013). In this study, we determined consistency with the cytoarchitectonic results by measuring the overlap ratio between our MPM results using connectivity-based parcellation and the results from FZ Jülich’s SPM Anatomy Toolbox. We also calculated the consistency between the results based on the local connectivity profiles and the results based on the resting-state fMRI data by measuring their overlap. We determined the stability by transforming the result for each individual subject into MNI space and then calculating the stability as the overlap ratio for each subject’s result with the MPM.

## Results

### Connectivity-Based Parcellation of the Primary Cerebral Cortices

**FIGURE 1:**
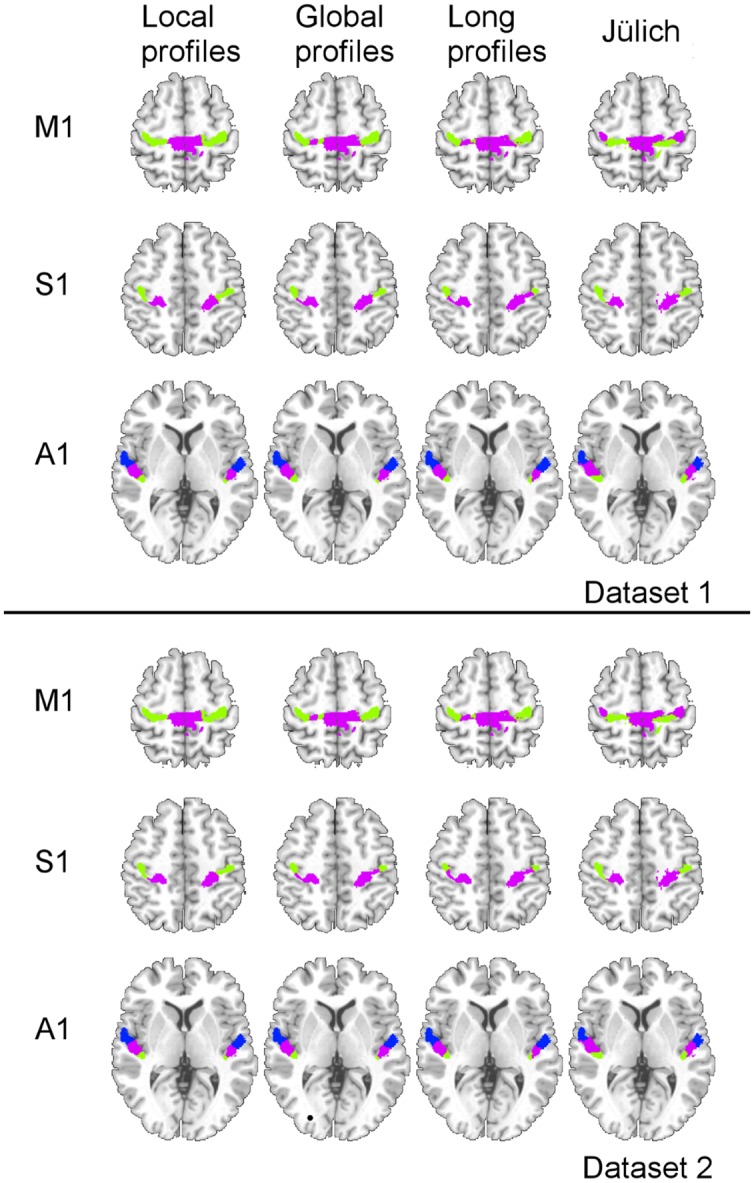
**Comparison of the MPM results using local, global, and long connectivity profiles with the cytoarchitectonic results from the SPM Anatomy Toolbox (FZ Jülich’s results)**.

**FIGURE 2:**
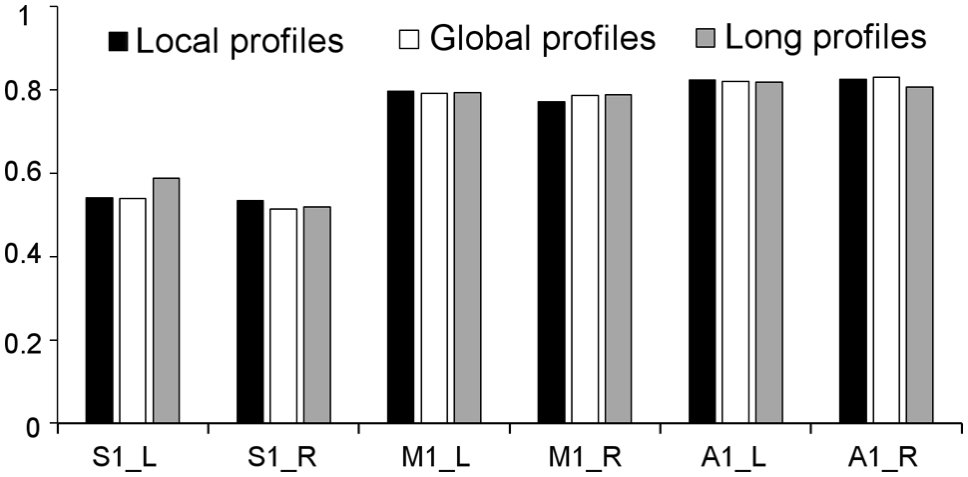
**Consistency between the results from dataset 1 using local, global, and long connectivity profiles and the cytoarchitectonic results from the SPM Anatomy Toolbox**.

Two subregions, the M1 anterior and M1 posterior, were identified within the M1 for all three different connectivity profiles using connectivity-based parcellation (**Figure 1**, M1). The parcellation results for both the independent datasets were consistent with the cytoarchitectonic subdivision of M1 (Geyer et al., 1996) from FZ Jülich’s SPM Anatomy Toolbox (Eickhoff et al., 2005; **Figure 1**, M1). Quantitatively, the overlap ratio between our identified regions and the SPM anatomy toolbox were 79.61, 79.25, and 79.41% in the left hemisphere and 77.10, 78.74, and 78.90% in the right hemisphere using the local, global, and long connectivity profiles in dataset 1, respectively (**Figure 2**). The A1 was divided into three subregions along the medio-lateral axis of Heschl’s gyrus (**Figure 1**, A1). The correspondence between our parcellation results and the cytoarchitectonic results (Morosan et al., 2001) from FZ Jülich’s SPM Anatomy Toolbox (Eickhoff et al., 2005) for A1 was good, as it had been for the M1. The consistency between the two results was 82.33, 82.13, and 81.96% in the left hemisphere and 82.63, 82.98, and 80.68% in the right hemisphere using the local, global and long connectivity profiles, respectively. The S1 was parcellated into two subregions, which were arranged medially to laterally (**Figure 1**, S1). Our results showed 54.11, 53.95, and 58.79% overlap in the left hemisphere and 53.36, 51.39, and 51.86% overlap in the right hemisphere using the local, global, and long connectivity profiles, respectively, with the cytoarchitectonic results (Schönwiesner et al., 2002; **Figure 2**). We parcellated the V1 into two areas, the anterior and posterior areas. The parcellation of the V1 was performed to ensure the completeness of our work on the parcellation of the primary cerebral cortices and was also used later in the validation of the stability of the local connectivity profiles. Our parcellation results for the M1, A1, S1, and V1 in dataset 2 showed a similar pattern to those from dataset 1 (Supplementary Figure S1).

### Resting-State fMRI based Parcellation of the Primary Cerebral Cortices

**FIGURE 3:**
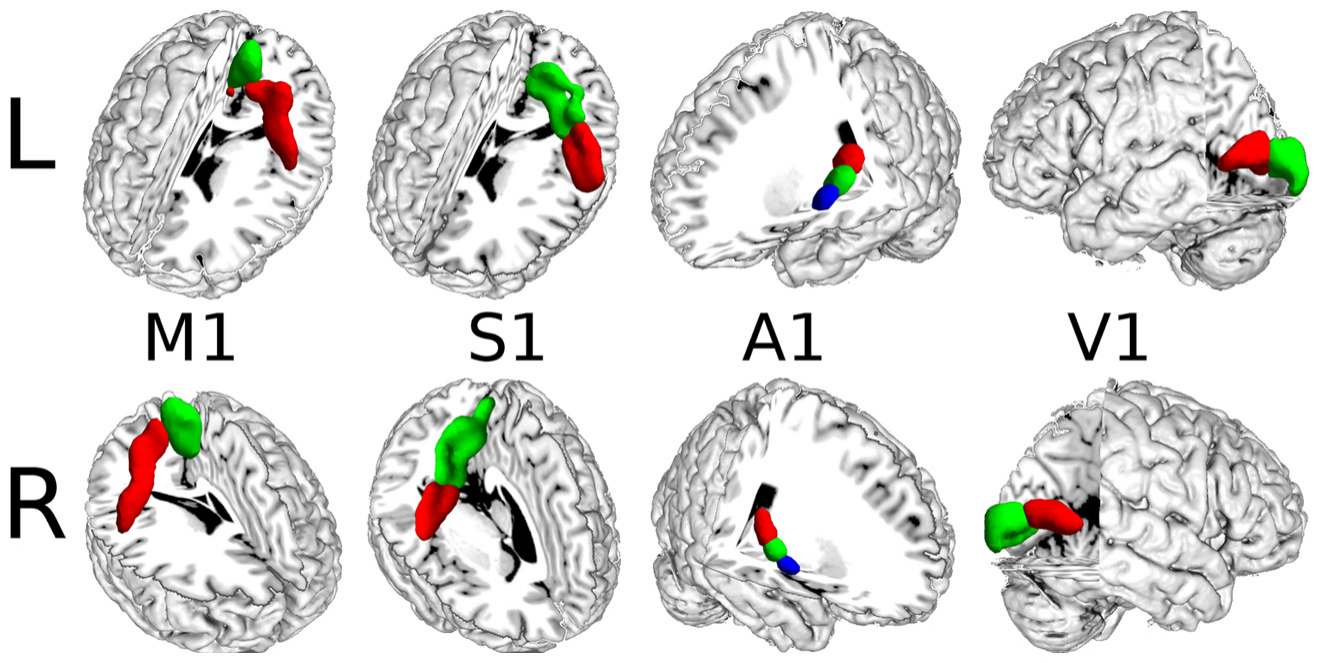
**The MPM results for the primary cerebral cortices based on resting-state fMRI**.

The results based on resting-state fMRI images (**Figure 3**) were highly consistent with the results obtained by connectivity-based parcellation using local connectivity profiles. The consistency was 96.74% for the left M1 and 98.96% for the right M1; 74.00% for the left S1 and 77.67% for the right S1; 94.82% for the left A1 and 90.00% for the right A1; and 95.45% for the left V1 and 92.94% for the right V1.

### Stability of the Three Parcellation Results

**FIGURE 4:**
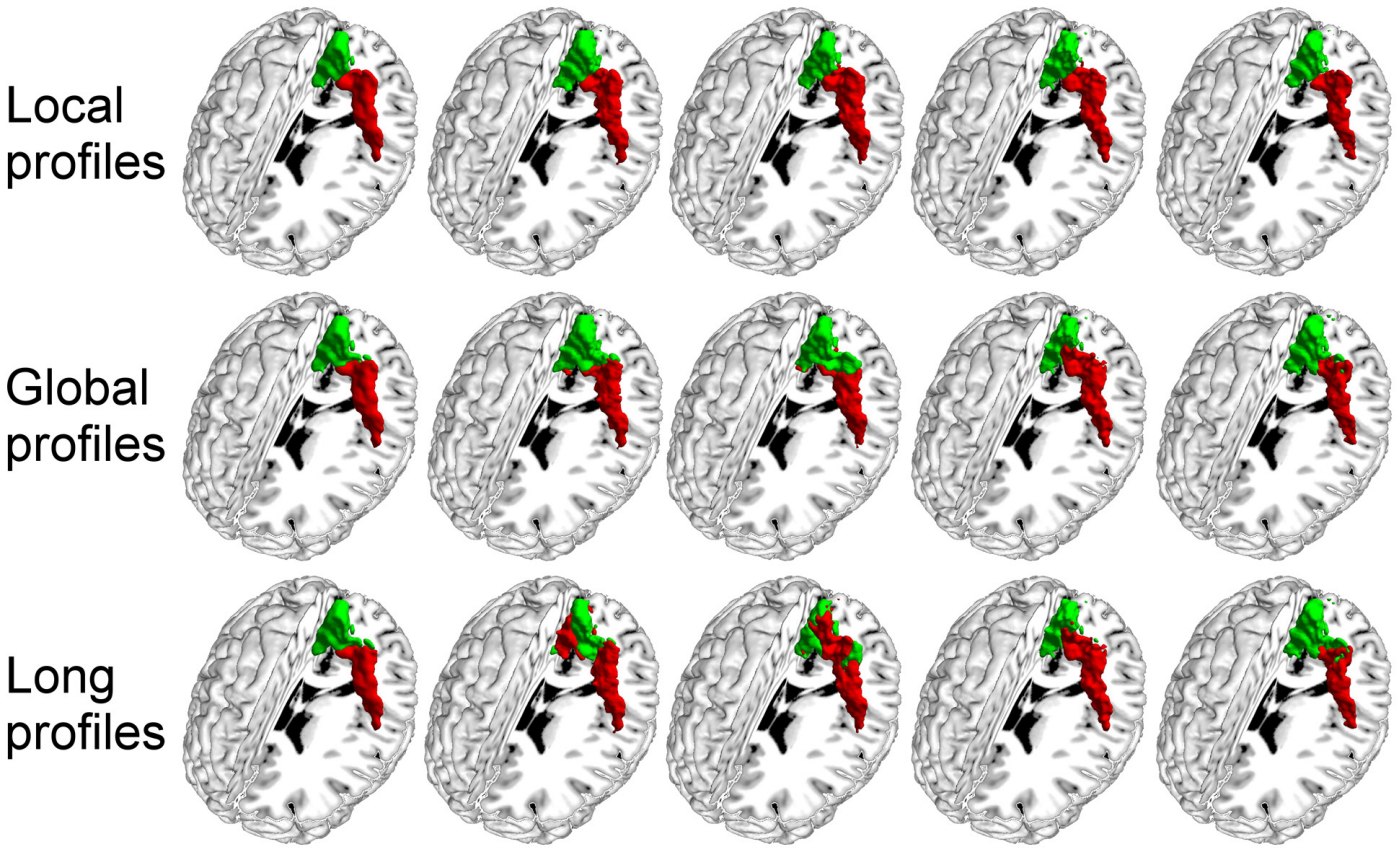
**The primary motor cortex (M1) in the left hemisphere was parcellated into two subregions using local, global, and long connectivity profiles.** The MPM from dataset 1 of the M1 (first column) and 4 individual example results using local, global, and long connectivity profiles are shown.

**FIGURE 5:**
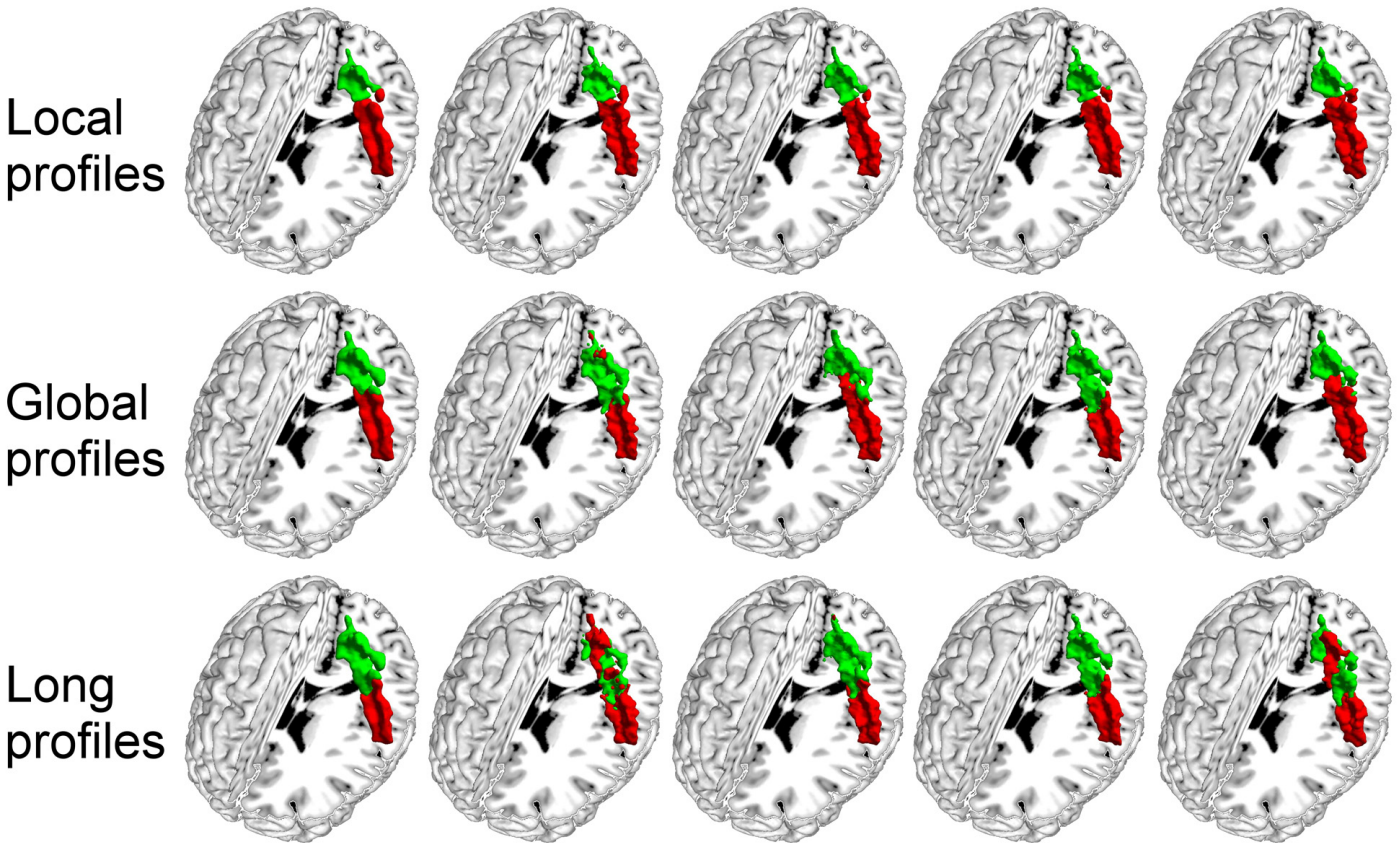
**The primary somatosensory cortex (S1) in the left hemisphere was parcellated into two subregions using local, global, and long connectivity profiles.** The MPM from dataset 1 of the S1 (first column) and four individual example results using local, global, and long connectivity profiles are shown.

**FIGURE 6:**
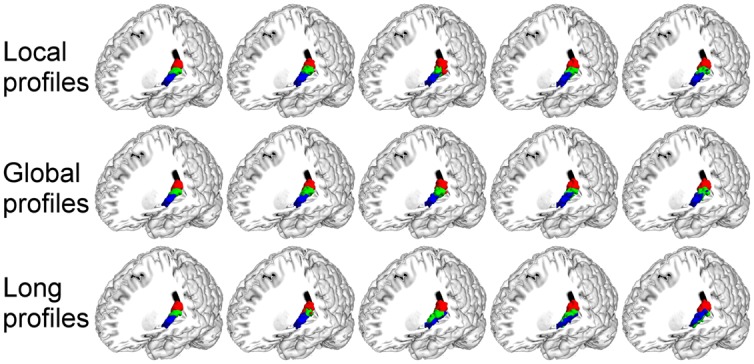
**The primary auditory cortex (A1) in the left hemisphere was parcellated into three subregions using local, global, and long connectivity profiles.** The MPM from dataset 1 of the A1 (first column) and four individual example results using local, global, and long connectivity profiles are shown.

**FIGURE 7:**
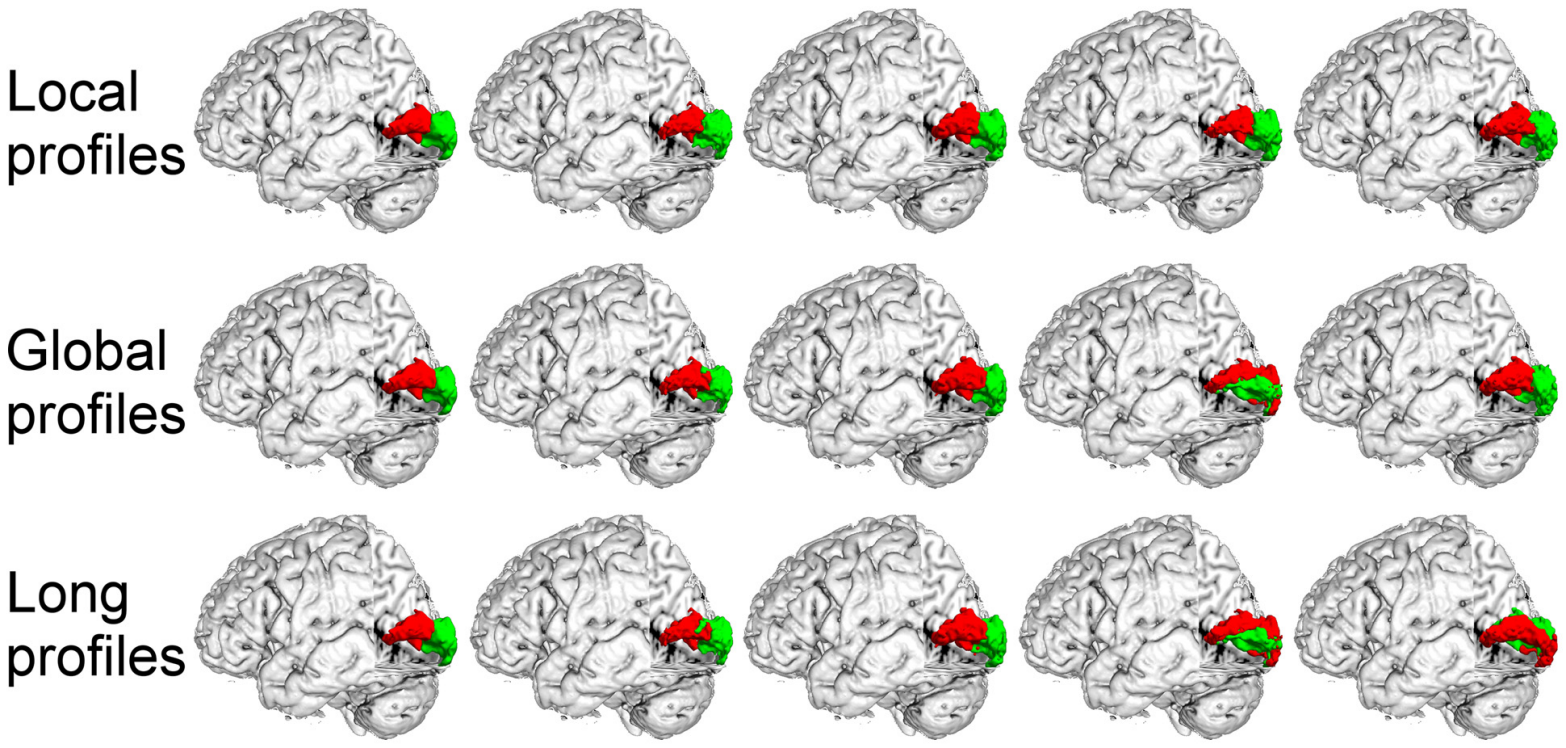
**The primary visual cortex (V1) in the left hemisphere was parcellated into two subregions using local, global, and long connectivity profiles.** The MPM from dataset 1 of the V1 (first column) and four individual example results using local, global and long connectivity profiles are shown.

**FIGURE 8:**
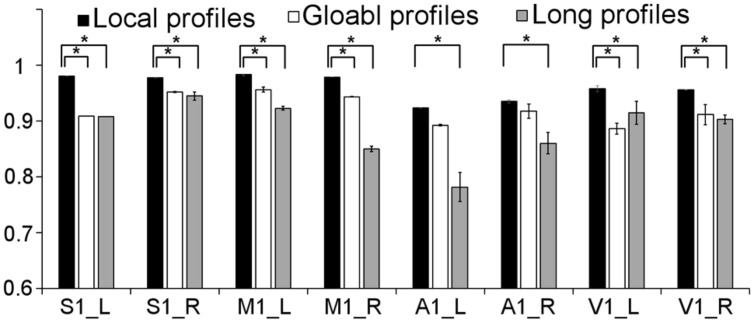
**Comparison of the stability between the results using local, global, and long connectivity profiles from dataset 1; groups with ^∗^ means that the differences were significant, *p* < 0.001; error bars indicate mean ± SD**.

We further compared the parcellation results using the local, global, and long connectivity profiles separately. The parcellation results of the group MPM and four randomly selected subjects from dataset 1 are shown (**Figure 4** for the left M1, **Figure 5** for the left S1, **Figure 6** for the left A1 and **Figure 7** for the left V1, Supplementary Figure S3 for the right M1, Supplementary Figure S4 for the right S1, Supplementary Figure S5 for the right A1, and Supplementary Figure S6 for the right V1). For the M1, V1, and S1, the borders of the subregions that were identified using local connectivity profiles exhibited significantly higher stability than the results obtained by using global and long connectivity profiles in both hemispheres for dataset 1 (Wilcoxon signed rank test, *p* < 0.001; **Figure 8**). For the A1, the parcellation results obtained using local connectivity profiles were more stable than the results obtained using long connectivity profiles, but no difference was found between the local and global connectivity profiles (*p* = 0.1942 for the left hemisphere, *p* = 0.7738 for the right hemisphere). In dataset 2, the borders of the subregions of all four primary cerebral cortices obtained using local connectivity profiles exhibited significant higher stability than the results obtained using global and long connectivity profiles in both hemispheres (Wilcoxon signed rank test, *p* < 0.001; Supplementary Figure S2), with an exception that there was no significant difference between the results using the local and global connectivity profiles for A1 in the right hemisphere (*p* = 0.0018).

## Discussion

In this study, we proposed that local connectivity profiles might be advantageous for parcellating the primary cerebral cortices (M1, S1, A1, and V1). Global, long, and local connectivity profiles were used to test this hypothesis. We found that the results of the three profiles were all quite consistent with the cytoarchitectonic results. More importantly, the results obtained using the local connectivity profiles showed less inter-subject variability than the results obtained using the other two. This finding suggests that local connectivity profiles are suitable for parcellating the primary cerebral cortices and superior to global and long connectivity profiles. This may also imply that different connectivity profiles should be adopted depending on the structural characteristics of different areas of the cerebral cortices.

Subdivision of the primary cerebral cortices is essential for improving our understanding of the human brain. Since the important milestone work, Brodmann’s map (Brodmann, 1909), was constructed, intensive research based on cytoarchitecture, myeloarchitecture, and functional brain imaging has been performed for the primary cerebral cortices. The M1 is primarily responsible for descending motor commands for voluntary movement. Many studies in both monkeys and humans have suggested that the M1 contains subdivisions. For example, Geyer et al. (1996) subdivided the M1 into the M1 anterior and the M1 posterior based on quantitative cytoarchitecture, quantitative distributions of transmitter-binding sites, and positron emission tomography. Rathelot and Strick (2009) found two subdivisions of the M1 in rhesus monkey, based on different distributions of cortico-motoneuronal cells. Nebel et al. (2014) used patterns of correlations in fMRI data to localize five functional subdivisions of the M1. However, previous research into the M1 was primarily based on post-mortem analysis or functional brain imaging. In this study, we subdivided the M1 into the M1 anterior and the M1 posterior (**Figure 1**, M1) using a non-invasive, connectivity-based parcellation method with local, global, and long connectivity profiles. The borders of the two subregions obtained using local connectivity profiles were almost the same as the results obtained using global and long connectivity profiles and corresponded well with previous results (Geyer et al., 1996). The consistency between our results and cytoarchitectonic results indicates that connectivity-based parcellation is appropriate for the M1.

The A1 is primarily responsible for processing auditory information in humans, and research has indicated that the A1 is tonotopically organized (Talavage et al., 2004), but the precise locations of these tonotopic areas remains unclear. Much research (Kaas and Hackett, 2000; Morosan et al., 2001) has been devoted to investigating the subdivisions of the A1 because of the good correspondence between these subdivisions and the tonotopic organization within it (Kaas and Hackett, 2000; Formisano et al., 2003). Currently, the human A1 is difficult to identify *in vivo* and the information about it has primarily been defined from post-mortem brains. For example, the A1 has been variously described as BA 41 (Brodmann, 1909), area TC (von Economo and Horn, 1930), or Te1 (Morosan et al., 2001). Additionally, the number of subdivisions of the A1 has varied between different studies (Iannetti et al., 2003; Sewards, 2011; Wasserthal et al., 2014). In this study, we chose the cytoarchitectonic map of Te1 in Morosan et al. (2001) as a seed ROI for the A1 and identified it as having three subregions (**Figure 1**, A1). The results obtained using local connectivity profiles were in good agreement with the results obtained using global and long connectivity profiles and were similar to the Te1.1, Te1.0, and Te1.2 in Morosan et al. (2001). Our parcellation results were also structurally in agreement with the functionally mirror image tonotopic organization of the A1, which consists of a high-low-high frequency gradient from the rostrolateral to the caudomedial direction (Formisano et al., 2003; Upadhyay et al., 2007; Langers and van Dijk, 2012). Thus, our parcellation of the A1 into three subregions based on the structural connectivity-based method is reasonable.

The S1 is primarily involved in processing tactile and nociceptive stimuli and is complex and hierarchically organized (Ploner et al., 2000). Identifying the subregions of the S1 should be helpful for studying brain representations of the body. Traditionally, the S1 has been described as an area consisting of 3a, 3b, 1, and 2 (Brodmann, 1909). In this study, we followed the recent definition by Keysers et al. (2010) that defined the S1 as BA 3, because of its homogeneity with the other sensory cortices. We found that the two subregions of the S1 (**Figure 1**, S1) identified using connectivity-based parcellation were different from Geyer’s results (Schönwiesner et al., 2002), a difference that may be attributable to our using different imaging modalities. Additionally, the BA 3a region is so small (Schönwiesner et al., 2002) that current low-resolution diffusion MRI brain images cannot parcellate it well. In any case, our results obtained using local connectivity profiles were still in accord with the results obtained using global and long connectivity profiles, and the medial-lateral organization was in good agreement with the somatotopic maps of the fingers that have been identified for 3b (Sanchez-Panchuelo et al., 2012, 2014). This finding suggests that connectivity-based parcellation is applicable to the S1.

Area V1 is primarily used to respond to the visual world and is thought to be relatively functionally monomodal. However, increasing evidence indicates that the V1 is a more complex and comprehensive area than previously thought (Petro et al., 2014). For example, Sugita (1999) and Lee and Nguyen (2001) found that the V1 could respond to stimuli that were not directly presented to the retina. Zhu et al. (2014) found that the V1 was hyperactive in individuals with post-traumatic stress disorder even during eyes-closed, resting-state fMRI scanning. Further, some recent studies suggested that the V1 may not be a single anatomic region. Yu et al. (2014) found that the anterior and posterior subregions of the V1 in patients with primary open-angle glaucoma differed in their changes in cortical thickness compared to the corresponding subregions of normal subjects. The FZ Jülich’s SPM Anatomy toolbox does not provide information about the subregions of the V1. In the present study, we used local, global, and long connectivity profiles to identify subdivisions of the V1. We found that the results obtained using the three types of profiles were highly consistent and were in line with the anterior-posterior pattern of the V1 provided by Boucard et al. (2009) and Yu et al. (2014). The results obtained in this current study may provide a new view of the V1.

Considering all the above evidence, we conclude that the parcellation results of the primary cerebral cortices obtained using local connectivity profiles correspond very well with the results obtained using global and long connectivity profiles as well as with the probabilistic cytoarchitectonically defined cortical areas.

In this study, the M1, A1, S1, and V1 were also parcellated using resting-state fMRI to test the viability of local connectivity profiles. The results (**Figure 3**) obtained from resting-state fMRI-based parcellation and the results obtained using local connectivity profiles-based parcellation corresponded very well. These findings further validate the viability of the local connectivity profiles-based parcellation.

The present study found that the borders of the subregions of the primary cerebral cortices obtained using local connectivity profiles showed less inter-subject variability compared to the borders of those obtained using global and long connectivity profiles (**Figure 8**). These findings have many potential explanations. First, convergent evidence indicates that the primary sensory and motor areas are heavily myelinated (Sigalovsky et al., 2006; Sereno et al., 2013; Glasser et al., 2014; Wasserthal et al., 2014). Some studies have suggested that horizontal fibers within the cortex consist primarily of local axonal ramifications of pyramidal neurons (Levitt et al., 1993). These horizontal fibers are important intracortical fibers between myelinated cells (Hellwig, 2002). Thus, it is reasonable for us to assume that the heavily myelinated primary cerebral cortices are rich in local axonal ramifications. From the above, we can infer that the primary cerebral cortices may be rich in local structure and function, which could lead to our finding local connectivity profiles, which are statistical descriptions of local axonal ramifications. On the other hand, the information processing steps of the primary cerebral cortices are fixed, which is beneficial for dealing with continuingly recurring standard tasks (Hellwig, 2002). This characteristic was partially confirmed by results using BOLD fMRI that showed that the primary cerebral cortices exhibited high local functional connections based on an intrinsic activity correlation (Sepulcre et al., 2010). Therefore, unlike the subcortical areas and the association cortex, the primary cerebral cortices may primarily exchange information that is constrained to local regions. Finally, the strength of the connectivity profiles obtained using tractography, as well as the certainty about the orientation measurements, decreases with distance between the source and target areas (Raj and Chen, 2011). Thus, local connectivity profiles, especially for the primary cerebral cortices, which are heavily myelinated and rich in local functional connectivity, may be less influenced by these deficiencies than global and long connectivity profiles. In summary, we therefore suggest that local connectivity profiles are more suitable for parcellating the primary cerebral cortices.

## Conclusion

We found that local connectivity profiles seemed to do a better job of parcellating the primary cortices. Prior to our study, many subcortical areas and the association cortex have been subdivided by connectivity-based parcellation using global or long connectivity profiles. Our findings and previous studies imply that, in order to obtain accurate and useful parcellations, frameworks should be adopted that correspond to the characteristics of the various regions of the cerebral cortices. Our work provides a new perspective for completing the parcellation framework.

## Conflict of Interest Statement

The authors declare that the research was conducted in the absence of any commercial or financial relationships that could be construed as a potential conflict of interest.

## Footnotes

1 http://fcon_1000.projects.nitrc.org/indi/enhanced/

2 http://www.fmrib.ox.ac.uk/fsl

3 http://www.fz-juelich.de/SharedDocs/Downloads/INM/INM-1/DE/Toolbox/Toolbox_20.html; jsessionid=0B82DC28FF06C133EF5ACAD6D841DE54?nn=563092

## References

[B1] Amunts K. Malikovic A. Mohlberg H. Schormann T. Zilles K. (2000). Brodmann’s areas 17 and 18 brought into stereotaxic space-where and how variable? *Neuroimage* 11 66–84 10.1006/nimg.1999.0516 10686118

[B2] Anwander A. Tittgemeyer M. Von Cramon D. Y. Friederici A. D. Knosche T. R. (2007). Connectivity-based parcellation of Broca’s Area. *Cereb. Cortex* 17 816–825 10.1093/cercor/bhk034 16707738

[B3] Beckmann M. Johansen-Berg H. Rushworth M. F. (2009). Connectivity-based parcellation of human cingulate cortex and its relation to functional specialization. *J. Neurosci.* 29 1175–1190 10.1523/jneurosci.3328-08.2009 19176826 PMC6665147

[B4] Behrens T. E. Berg H. J. Jbabdi S. Rushworth M. F. Woolrich M. W. (2007). Probabilistic diffusion tractography with multiple fibre orientations: what can we gain? *Neuroimage* 34 144–155 10.1016/j.neuroimage.2006.09.018 17070705 PMC7116582

[B5] Binkofski F. Fink G. R. Geyer S. Buccino G. Gruber O. Shah N. J. (2002). Neural activity in human primary motor cortex areas 4a and 4p is modulated differentially by attention to action. *J. Neurophysiol.* 88 514–519. 12091573 10.1152/jn.2002.88.1.514

[B6] Blankenburg F. Ruben J. Meyer R. Schwiemann J. Villringer A. (2003). Evidence for a rostral-to-caudal somatotopic organization in human primary somatosensory cortex with mirror-reversal in areas 3b and 1. *Cereb. Cortex* 13 987–993 10.1093/cercor/13.9.987 12902398

[B7] Boucard C. C. Hernowo A. T. Maguire R. P. Jansonius N. M. Roerdink J. B. Hooymans J. M. (2009). Changes in cortical grey matter density associated with long-standing retinal visual field defects. *Brain* 132 1898–1906 10.1093/brain/awp119 19467992 PMC2702836

[B8] Brodmann K. (1909). *Vergleichende Lokalisationslehre der Groβhirnrinde in ihren Prinzipien dargestellt auf Grund des Zellenbaues*. Leipzig, Barth, JA.

[B9] Cloutman L. L. Lambon Ralph M. A. (2012). Connectivity-based structural and functional parcellation of the human cortex using diffusion imaging and tractography. *Front. Neuroanat.* 6:34 10.3389/fnana.2012.00034 PMC3429885 22952459

[B10] Eickhoff S. B. Stephan K. E. Mohlberg H. Grefkes C. Fink G. R. Amunts K. (2005). A new SPM toolbox for combining probabilistic cytoarchitectonic maps and functional imaging data. *Neuroimage* 25 1325–1335 10.1016/j.neuroimage.2004.12.034 15850749

[B11] Fan L. Wang J. Zhang Y. Han W. Yu C. Jiang T. (2014). Connectivity-based parcellation of the human temporal pole using diffusion tensor imaging. *Cereb. Cortex* 24 3365–3378 10.1093/cercor/bht196 23926116

[B12] Felleman D. J. Van Essen D. C. (1991). Distributed hierarchical processing in the primate cerebral cortex. *Cereb. Cortex* 1 1–47 10.1093/cercor/1.1.1 1822724

[B13] Formisano E. Kim D. S. Di Salle F. Van De Moortele P. F. Ugurbil K. Goebel R. (2003). Mirror-symmetric tonotopic maps in human primary auditory cortex. *Neuron* 40 859–869 10.1016/S0896-6273(03)00669-X 14622588

[B14] Galaburda A. Sanides F. (1980). Cytoarchitectonic organization of the human auditory cortex. *J. Comp. Neurol.* 190 597–610 10.1002/cne.901900312 6771305

[B15] Geyer S. Ledberg A. Schleicher A. Kinomura S. Schormann T. Burgel U. (1996). Two different areas within the primary motor cortex of man. *Nature* 382 805–807 10.1038/382805a0 8752272

[B16] Geyer S. Schormann T. Mohlberg H. Zilles K. (2000). Areas 3a, 3b, and 1 of human primary somatosensory cortex. Part 2. Spatial normalization to standard anatomical space. *Neuroimage* 11 684–696 10.1006/nimg.2000.0548 10860796

[B17] Glasser M. F. Goyal M. S. Preuss T. M. Raichle M. E. Van Essen D. C. (2014). Trends and properties of human cerebral cortex: correlations with cortical myelin content. *Neuroimage* 93(Pt 2), 165–175 10.1016/j.neuroimage.2013.03.060 23567887 PMC3795824

[B18] Glasser M. F. Van Essen D. C. (2011). Mapping human cortical areas in vivo based on myelin content as revealed by T1- and T2-weighted MRI. *J. Neurosci.* 31 11597–11616 10.1523/jneurosci.2180-11.2011 21832190 PMC3167149

[B19] Hellwig B. (2002). “Simple rules relate the cyto- and myeloarchitectonics of the human cerebral cortex: uniformity in areal diversity,” in *Cortical Areas: Unity and Diversity* eds Schu¨ Z. A. Miller R. (London: Taylor & Francis) 20–28.

[B20] Iannetti G. D. Porro C. A. Pantano P. Romanelli P. L. Galeotti F. Cruccu G. (2003). Representation of different trigeminal divisions within the primary and secondary human somatosensory cortex. *Neuroimage* 19 906–912 10.1016/S1053-8119(03)00139-3 12880819

[B21] Jenkinson M. Smith S. (2001). A global optimisation method for robust affine registration of brain images. *Med. Image Anal.* 5 143–156 10.1016/S1361-8415(01)00036-6 11516708

[B22] Johansen-Berg H. Behrens T. E. Robson M. D. Drobnjak I. Rushworth M. F. Brady J. M. (2004). Changes in connectivity profiles define functionally distinct regions in human medial frontal cortex. *Proc. Natl. Acad. Sci. U.S.A.* 101 13335–13340 10.1073/pnas.0403743101 15340158 PMC516567

[B23] Kaas J. H. Hackett T. A. (2000). Subdivisions of auditory cortex and processing streams in primates. *Proc. Natl. Acad. Sci. U.S.A.* 97 11793–11799 10.1073/pnas.97.22.11793 11050211 PMC34351

[B24] Kahnt T. Chang L. J. Park S. Q. Heinzle J. Haynes J. D. (2012). Connectivity-based parcellation of the human orbitofrontal cortex. *J. Neurosci.* 32 6240–6250 10.1523/jneurosci.0257-12.2012 22553030 PMC6622144

[B25] Keysers C. Kaas J. H. Gazzola V. (2010). Somatosensation in social perception. *Nat. Rev. Neurosci.* 11 417–428 10.1038/nrn2833 20445542

[B26] Klein J. C. Behrens T. E. Robson M. D. Mackay C. E. Higham D. J. Johansen-Berg H. (2007). Connectivity-based parcellation of human cortex using diffusion MRI: establishing reproducibility, validity and observer independence in BA 44/45 and SMA/pre-SMA. *Neuroimage* 34 204–211 10.1016/j.neuroimage.2006.08.022 17023184

[B27] Knosche T. R. Tittgemeyer M. (2011). The role of long-range connectivity for the characterization of the functional-anatomical organization of the cortex. *Front. Syst. Neurosci.* 5:58 10.3389/fnsys.2011.00058 PMC3133730 21779237

[B28] Langers D. R. van Dijk P. (2012). Mapping the tonotopic organization in human auditory cortex with minimally salient acoustic stimulation. *Cereb. Cortex* 22 2024–2038 10.1093/cercor/bhr282 21980020 PMC3412441

[B29] Lee T. S. Nguyen M. (2001). Dynamics of subjective contour formation in the early visual cortex. *Proc. Natl. Acad. Sci. U.S.A.* 98 1907–1911 10.1073/pnas.031579998 11172049 PMC29355

[B30] Levitt J. B. Lewis D. A. Yoshioka T. Lund J. S. (1993). Topography of pyramidal neuron intrinsic connections in macaque monkey prefrontal cortex (areas 9 and 46). *J. Comp. Neurol.* 338 360–376 10.1002/cne.903380304 8113445

[B31] Li W. Qin W. Liu H. Fan L. Wang J. Jiang T. (2013). Subregions of the human superior frontal gyrus and their connections. *Neuroimage* 78 46–58 10.1016/j.neuroimage.2013.04.011 23587692

[B32] Mars R. B. Jbabdi S. Sallet J. O’reilly J. X. Croxson P. L. Olivier E. (2011). Diffusion-weighted imaging tractography-based parcellation of the human parietal cortex and comparison with human and macaque resting-state functional connectivity. *J. Neurosci.* 31 4087–4100 10.1523/jneurosci.5102-10.2011 21411650 PMC3091022

[B33] Mesulam M. M. (1990). Large-scale neurocognitive networks and distributed processing for attention, language, and memory. *Ann. Neurol.* 28 597–613 10.1002/ana.410280502 2260847

[B34] Mesulam M. M. (2008). Representation, inference, and transcendent encoding in neurocognitive networks of the human brain. *Ann. Neurol.* 64 367–378 10.1002/ana.21534 18991346

[B35] Morosan P. Rademacher J. Schleicher A. Amunts K. Schormann T. Zilles K. (2001). Human primary auditory cortex: cytoarchitectonic subdivisions and mapping into a spatial reference system. *Neuroimage* 13 684–701 10.1006/nimg.2000.0715 11305897

[B36] Nebel M. B. Joel S. E. Muschelli J. Barber A. D. Caffo B. S. Pekar J. J. (2014). Disruption of functional organization within the primary motor cortex in children with autism. *Hum. Brain Mapp.* 35 567–580 10.1002/hbm.22188 23118015 PMC3864146

[B37] Ng A. Y. Jordan M. I. Weiss Y. (2002). “On spectral clustering: analysis and an algorithm,” in *Advances in Neural Information Processing Systems (ANIPS)* Vol. 14 eds Dietterich T. Becker S. Ghahramani Z. (Cambridge, MIT Press) 849–856 10.3389/fnins.2012.00152

[B38] Nooner K. B. Colcombe S. J. Tobe R. H. Mennes M. Benedict M. M. Moreno A. L. (2012). The NKI-Rockland sample: a model for accelerating the pace of discovery science in psychiatry. *Front. Neurosci.* 6:152 10.3389/fnins.2012.00152 PMC3472598 23087608

[B39] O’Leary D. D. Chou S. J. Sahara S. (2007). Area patterning of the mammalian cortex. *Neuron* 56 252–269 10.1016/j.neuron.2007.10.010 17964244

[B40] Petro L. S. Vizioli L. Muckli L. (2014). Contributions of cortical feedback to sensory processing in primary visual cortex. *Front. Psychol.* 5:1223 10.3389/fpsyg.2014.01223 PMC4222340 25414677

[B41] Ploner M. Schmitz F. Freund H. J. Schnitzler A. (2000). Differential organization of touch and pain in human primary somatosensory cortex. *J. Neurophysiol.* 83 1770–1776 10.1371/journal.pone.0014832 10712498

[B42] Raj A. Chen Y. H. (2011). The wiring economy principle: connectivity determines anatomy in the human brain. *PLoS ONE* 6:e14832 10.1371/journal.pone.0014832 PMC3168442 21915250

[B43] Rathelot J. A. Strick P. L. (2009). Subdivisions of primary motor cortex based on cortico-motoneuronal cells. *Proc. Natl. Acad. Sci. U.S.A.* 106 918–923 10.1073/pnas.0808362106 19139417 PMC2621250

[B44] Sanchez-Panchuelo R. M. Besle J. Beckett A. Bowtell R. Schluppeck D. Francis S. (2012). Within-digit functional parcellation of Brodmann areas of the human primary somatosensory cortex using functional magnetic resonance imaging at 7 tesla. *J. Neurosci.* 32 15815–15822 10.1523/jneurosci.2501-12.2012 23136420 PMC6621625

[B45] Sanchez-Panchuelo R. M. Besle J. Mougin O. Gowland P. Bowtell R. Schluppeck D. (2014). Regional structural differences across functionally parcellated Brodmann areas of human primary somatosensory cortex. *Neuroimage* 93(Pt 2) 221–230 10.1016/j.neuroimage.2013.03.044 23558101

[B46] Schönwiesner M. Von Cramon D. Y. Rübsamen R. (2002). Is it tonotopy after all? *Neuroimage* 17 1144–1161 10.1006/nimg.2002.1250 12414256

[B47] Sepulcre J. Liu H. Talukdar T. Martincorena I. Yeo B. T. Buckner R. L. (2010). The organization of local and distant functional connectivity in the human brain. *PLoS Comput. Biol.* 6:e1000808 10.1371/journal.pcbi.1000808 PMC2883589 20548945

[B48] Sereno M. I. Lutti A. Weiskopf N. Dick F. (2013). Mapping the human cortical surface by combining quantitative T(1) with retinotopy. *Cereb. Cortex* 23 2261–2268 10.1093/cercor/bhs213 22826609 PMC3729202

[B49] Sewards T. V. (2011). Adolf Hopf’s 1954 myeloarchitectonic parcellation of the human temporal lobe: a review and assessment. *Brain Res. Bull.* 86 298–313 10.1016/j.brainresbull.2011.08.010 21888952

[B50] Sigalovsky I. S. Fischl B. Melcher J. R. (2006). Mapping an intrinsic MR property of gray matter in auditory cortex of living humans: a possible marker for primary cortex and hemispheric differences. *Neuroimage* 32 1524–1537 10.1016/j.neuroimage.2006.05.023 16806989 PMC1839042

[B51] Sugita Y. (1999). Grouping of image fragments in primary visual cortex. *Nature* 401 269–272 10.1038/45785 10499583

[B52] Talavage T. M. Sereno M. I. Melcher J. R. Ledden P. J. Rosen B. R. Dale A. M. (2004). Tonotopic organization in human auditory cortex revealed by progressions of frequency sensitivity. *J. Neurophysiol.* 91 1282–1296 10.1152/jn.01125.2002 14614108

[B53] Triarhou L. C. (2007). A proposed number system for the 107 cortical areas of Economo and Koskinas, and Brodmann area correlations. *Stereotact. Funct. Neurosurg.* 85 204–215 10.1159/000103259 17534133

[B54] Upadhyay J. Ducros M. Knaus T. A. Lindgren K. A. Silver A. Tager-Flusberg H. (2007). Function and connectivity in human primary auditory cortex: a combined fMRI and DTI study at 3 Tesla. *Cereb. Cortex* 17 2420–2432 10.1093/cercor/bhl150 17190967

[B55] von Economo C. Horn L. (1930). Über Windungsrelief, Maße und Rindenarchitektonik der Supratemporalfläche, ihre individuellen und ihre Seitenunterschiede. *Z. Neurol. Psychiatry* 130 678–757.

[B56] Wang J. Fan L. Zhang Y. Liu Y. Jiang D. Zhang Y. (2012). Tractography-based parcellation of the human left inferior parietal lobule. *Neuroimage* 63 641–652 10.1016/j.neuroimage.2012.07.045 22846658

[B57] Wasserthal C. Brechmann A. Stadler J. Fischl B. Engel K. (2014). Localizing the human primary auditory cortex in vivo using structural MRI. *Neuroimage* 93(Pt 2) 237–251 10.1016/j.neuroimage.2013.07.046 23891882 PMC3902056

[B58] Yu L. Yin X. Dai C. Liang M. Wei L. Li C. (2014). Morphologic changes in the anterior and posterior subregions of V1 and V2 and the V5/MT+ in patients with primary open-angle glaucoma. *Brain Res.* 1588 135–143 10.1016/j.brainres.2014.09.005 25199592

[B59] Zhu H. Zhang J. Zhan W. Qiu C. Wu R. Meng Y. (2014). Altered spontaneous neuronal activity of visual cortex and medial anterior cingulate cortex in treatment-naive posttraumatic stress disorder. *Compr. Psychiatry* 55 1688–1695 10.1016/j.comppsych.2014.06.009 25060989

