# Supplementary material for: Parcellation of the primary cerebral cortices based on local connectivity profiles
Source: Front Neuroanat. 2015 Apr 27;9:50. doi: 10.3389/fnana.2015.00050 (PMC4410601; doi:10.3389/fnana.2015.00050)
Supplement: Supplementary file 1 [file Data_Sheet_1.DOCX]

***Supplementary Material***

**Parcellation of primary cerebral cortices based on local connectivity profiles**

**Qiaojun Li^1,2^, Ming Song^1,2^, Lingzhong Fan^1,2^, Yong Liu^1,2^, Tianzi Jiang^1,2,3,4*^**

^­^_­_^1^Brainnetome Center, Institute of Automation, Chinese Academy of Sciences, Beijing, P. R. China

^2^National Laboratory of Pattern Recognition, Institute of Automation, Chinese Academy of Sciences, Beijing, P. R. China

^3^CAS Center for Excellence in Brain Science, Institute of Automation, Chinese Academy of Sciences, Beijing,

P. R. China

^4^The Queensland Brain Institute, University of Queensland, Brisbane, QLD, Australia

***Correspondence:** Tianzi Jiang, National Laboratory of Pattern Recognition, Institute of Automation, Chinese Academy of Sciences, Beijing 100190, P. R. China;

E-mail: [jiangtz@nlpr.ia.ac.cn](mailto:jiangtz@nlpr.ia.ac.cn); Tel: +86 10 8254 4778; Fax: +86 10 8254 4777

1. **Supplementary Figures and Tables**


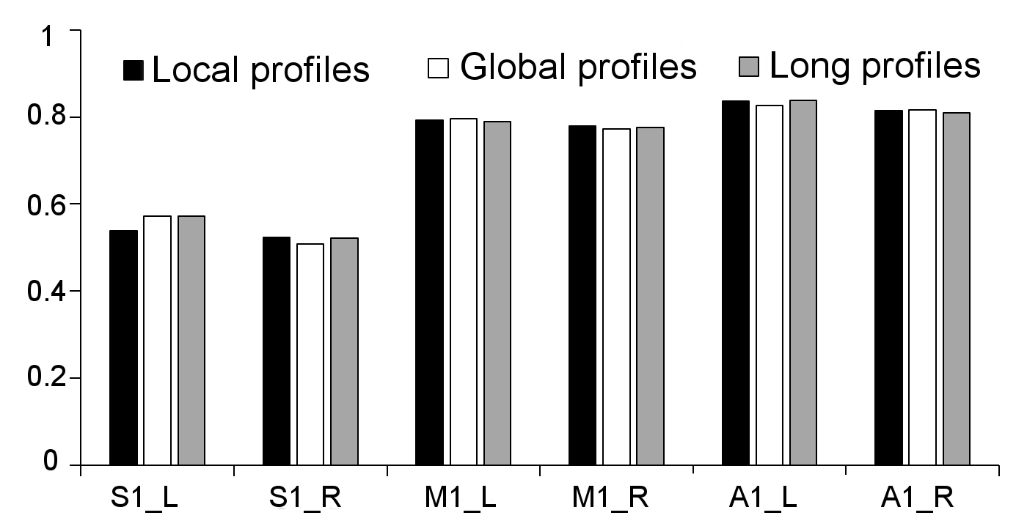


**FIGURE S1** Consistency between results using local, global and long connectivity profiles with cytoarchitectonic results from the SPM Anatomy Toolbox on dataset 2.


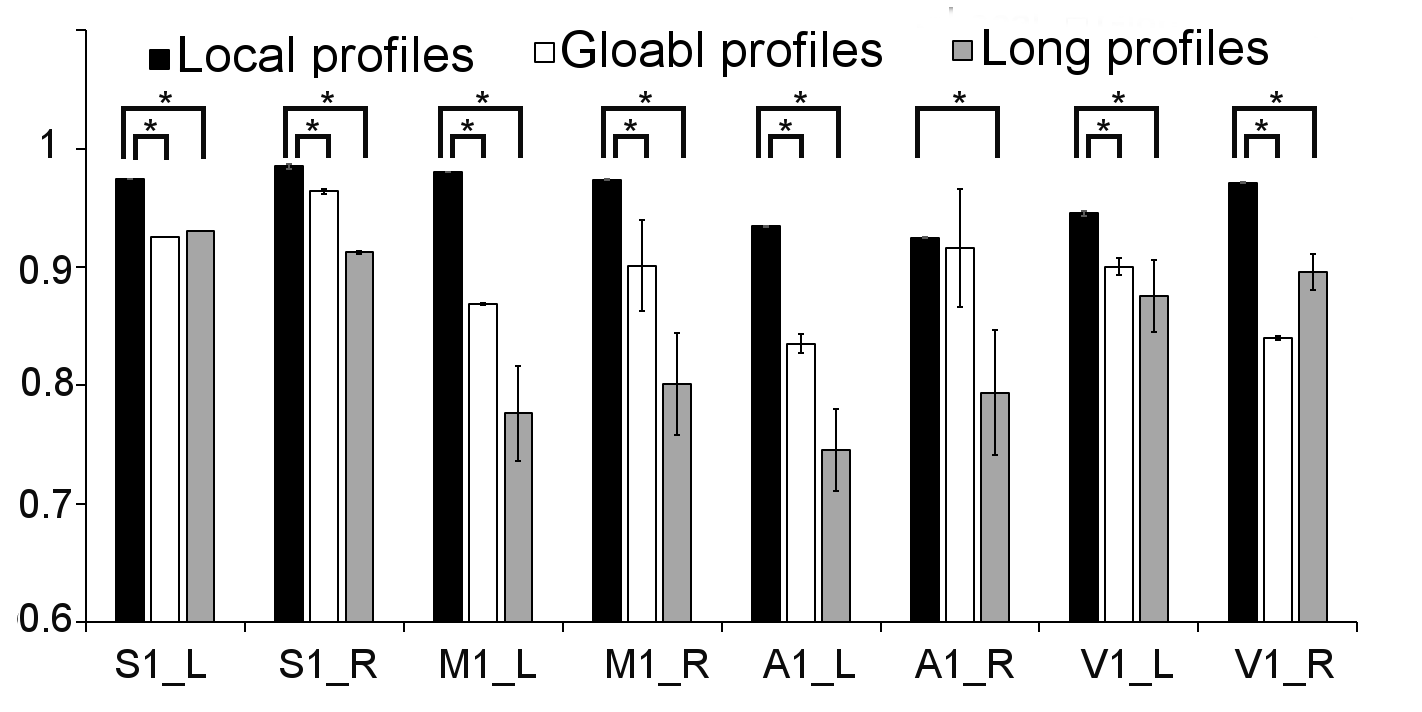


**FIGURE S2** Comparison of the stability between the results obtained using local, global, and long connectivity profiles on dataset 2, groups with * indicate a significant difference, Wilcoxon signed rank test, *p* < 0.001 between the pairs.


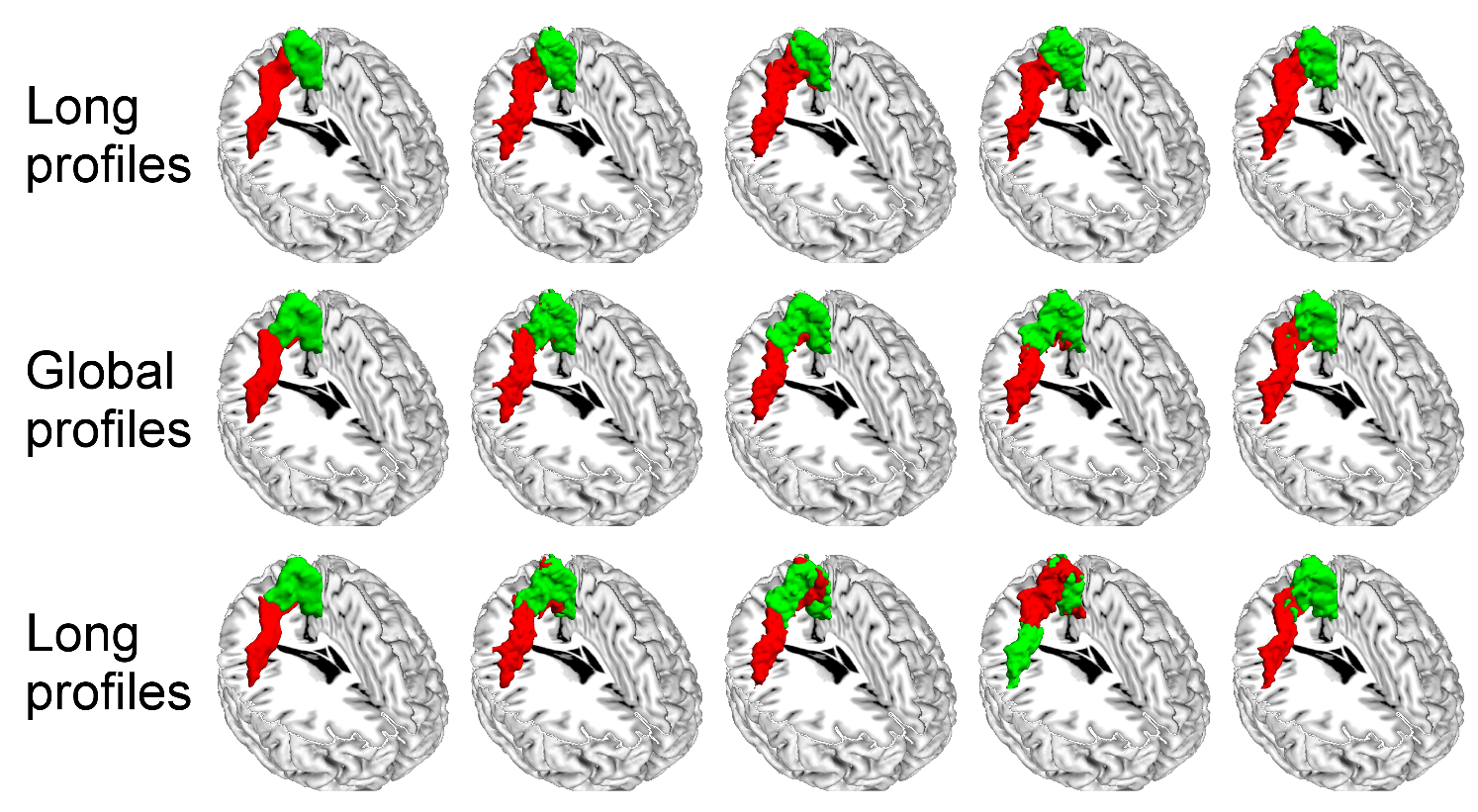


**FIGURE S3** The primary motor cortex (M1) in the right hemisphere was parceled into 2 subregions using local, global, and long connectivity profiles. A maximum probability map (MPM) of the M1 (first column) and 4 individual example results using local, global, and long connectivity profiles for dataset 1 are shown.


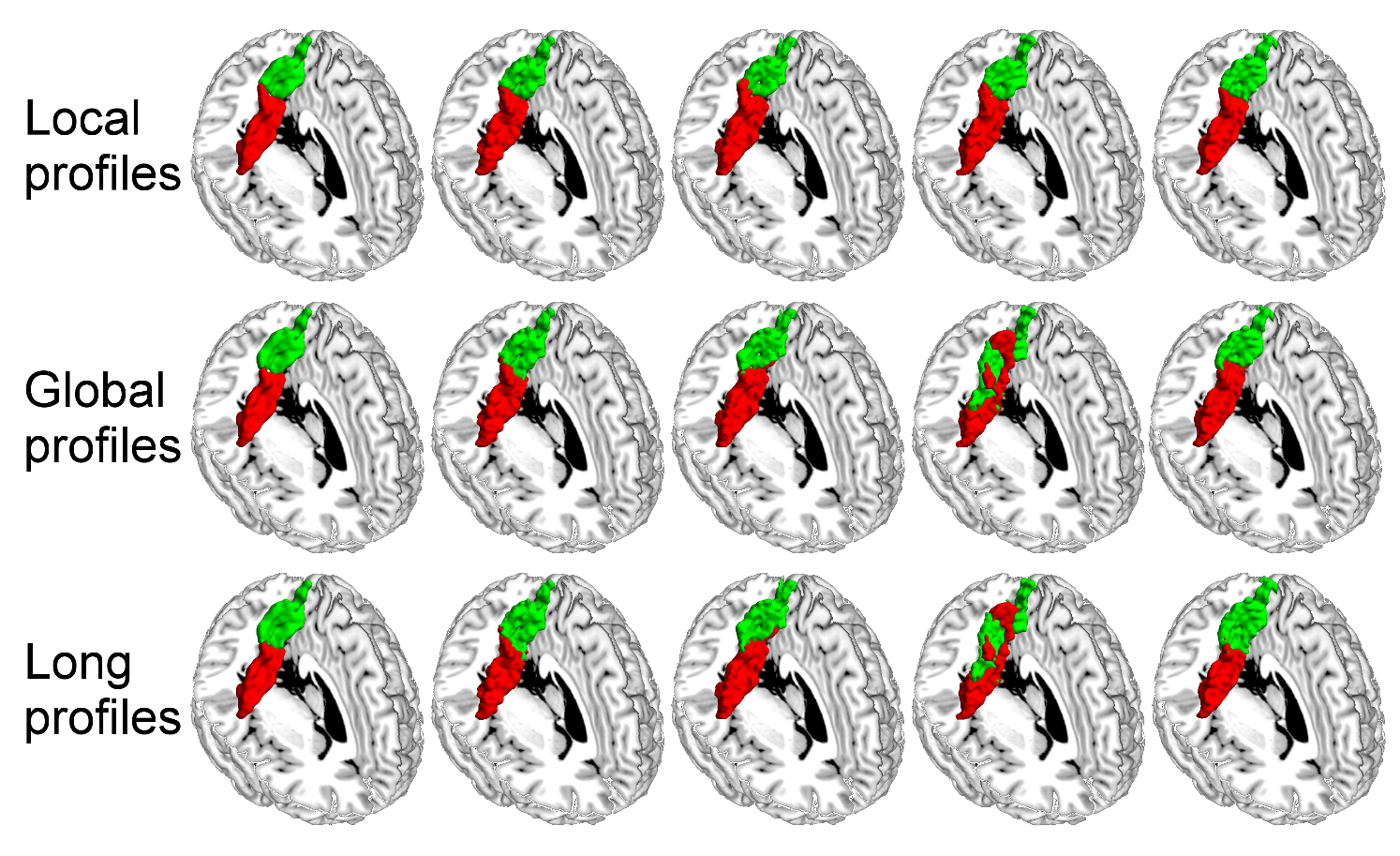


**FIGURE S4** The primary somatosensory cortex (S1) in the right hemisphere was parceled into 2 subregions using local, global, and long connectivity profiles. An MPM of the S1 (first column) and 4 individual example results using local, global and long connectivity profiles for dataset 1are shown.


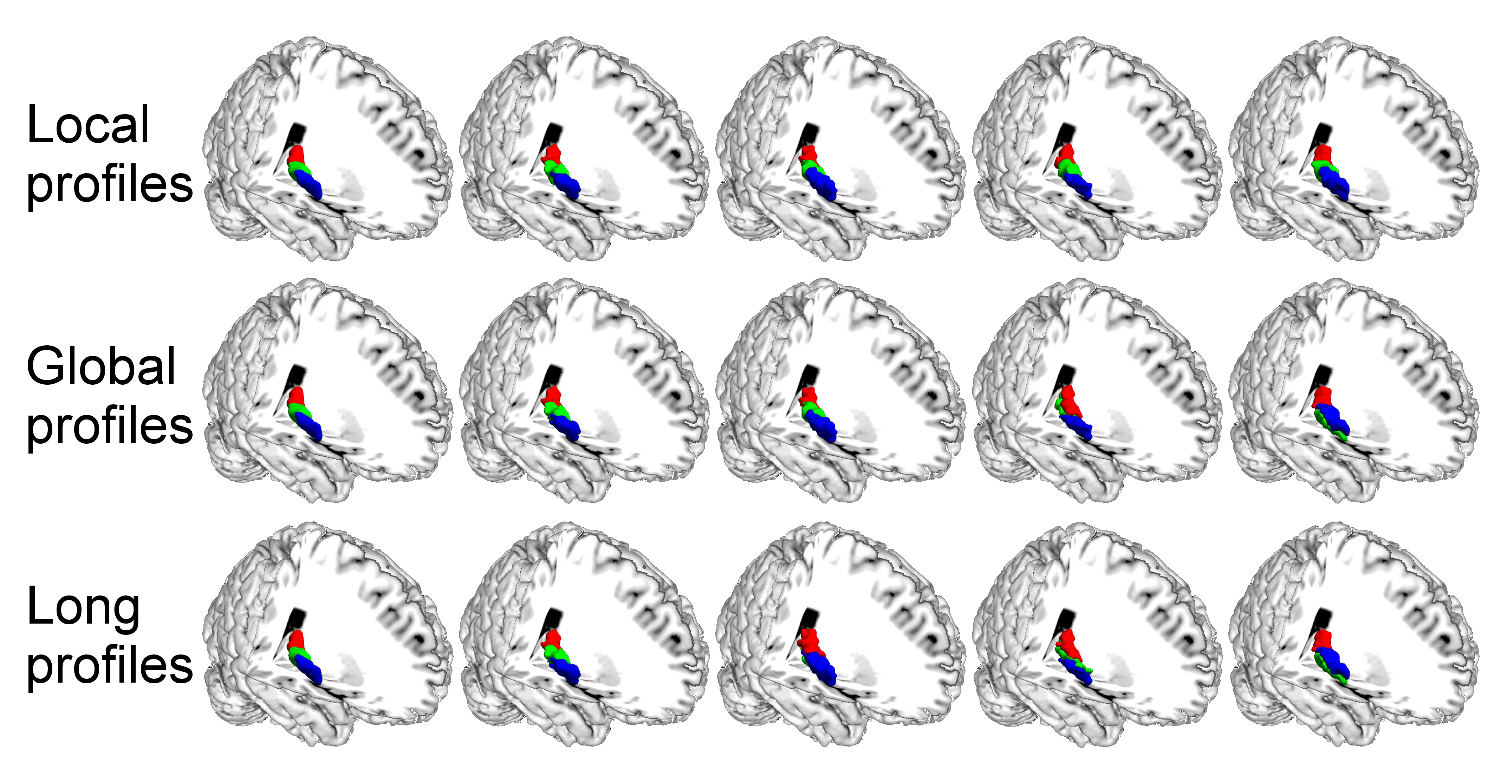


**FIGURE S5** The primary auditory cortex (A1) in the right hemisphere was parceled into 3 subregions using local, global, and long connectivity profiles. An MPM of the A1 (first column) and 4 individual example results using local, global, and long connectivity profiles for dataset 1 are shown.


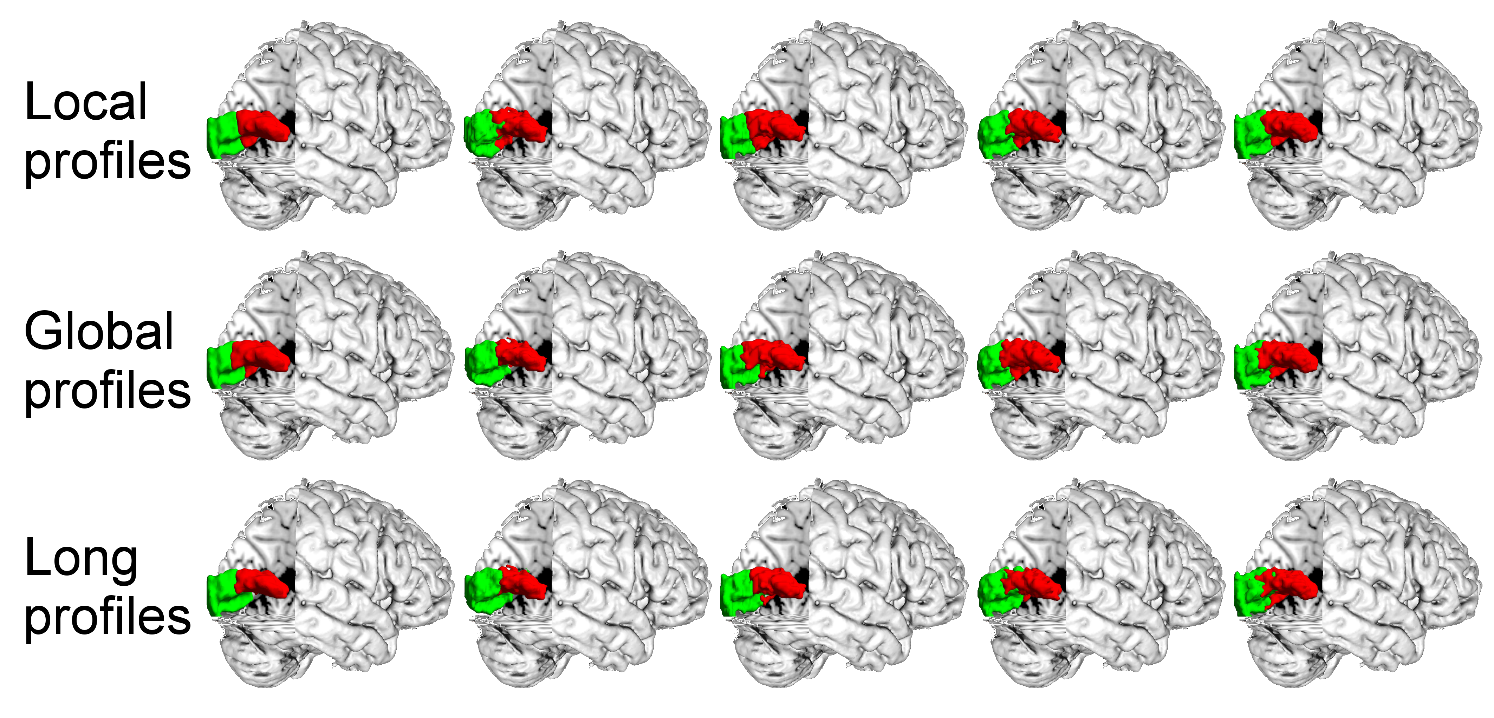


**FIGURE S6** The primary visual cortex (V1) in the right hemisphere was parceled into 2 subregions using local, global, and long connectivity profiles. An MPM of the V1 (first column) and 4 individual example results using local, global, and long connectivity profiles for dataset 1 are shown.

1. **Parcellation of the MT based on connectivity profiles using diffusion MRI**
   1. **Seed MT definition**

We parcellated the MT of the left hemisphere. To define the MT, Freesurfer's (version 5.3) automated recon-all pipeline was used on dataset 1 to parcellate the cortical and subcortical regions for each of the 20 subjects ([Fischl et al., 2004](#_ENREF_1)). In this study, the lh.MT.thresh.label in ‘fsaverage’ was used. After the carefully checking the accuracy of the segmentation, each subject’s MT mask was warped into MNI space and a MPM was calculated for the 20 masks using a threshold value of 0.5. This was defined as the MT seed mask. The 3D visualization of the mask is showed in **Figure R1 A**.

- 1. **Parcellation of the MT**

The MT was parcellated using the steps in the Materials and Methods section. In this study, we tried to parcellate the MT into 2 subdivisons using the local, global, and long connectivity profiles. As seen in the supplementary **Figure R1 B**, the MT was parcellated into dorsal and ventral subdivisions. The results from the three kinds of connectivity profiles were quite consistent. Also, the results using the local, global, and long connectivity profiles were highly stable, that is, they were 0.8063±0.1210, 0.7987±0.1004, and 0.7863±0.1140 for the respective results. There was no difference between the results using the local connectivity profiles and the global connectivity profiles (*p* = 0.9039) or between the results using local connectivity profiles and long connectivity profiles (*p* = 0.4939).

- 1. **Discussion of the MT results**

To date, no research about the parcellation of the MT has been published, as far as we are aware. However, some indirect evidence for the further subdivision of MT does exist. [Malikovic et al. (2007)](#_ENREF_3) stated that there was a small possibility that there was a correspondence between the 2 subregions of the hOc5 and the functionally defined MT and MST. Further, [Huk et al. (2002)](#_ENREF_2) reported that only part of the MT was activated. Thus, [Malikovic et al. (2007)](#_ENREF_3) thought that subregions of the MT may exist. What is more, different parts of the MT correspond to central and peripheral representations of the visual field ([Huk et al., 2002](#_ENREF_2)), that is, the ventral extreme of the MT is primarily responsible for the central representation and the dorsal and/or anterior borders of the MT are primarily responsible for peripheral stimulation. Therefore, subregions of the MT may exist. Although our parcellations of the MT cannot, therefore, be used to confirm the validity of our use of local connectivity profiles, the fact that we found consistency between the MT results for local, global, and long connectivity profiles, does seem to provide evidence for the validity of this method.


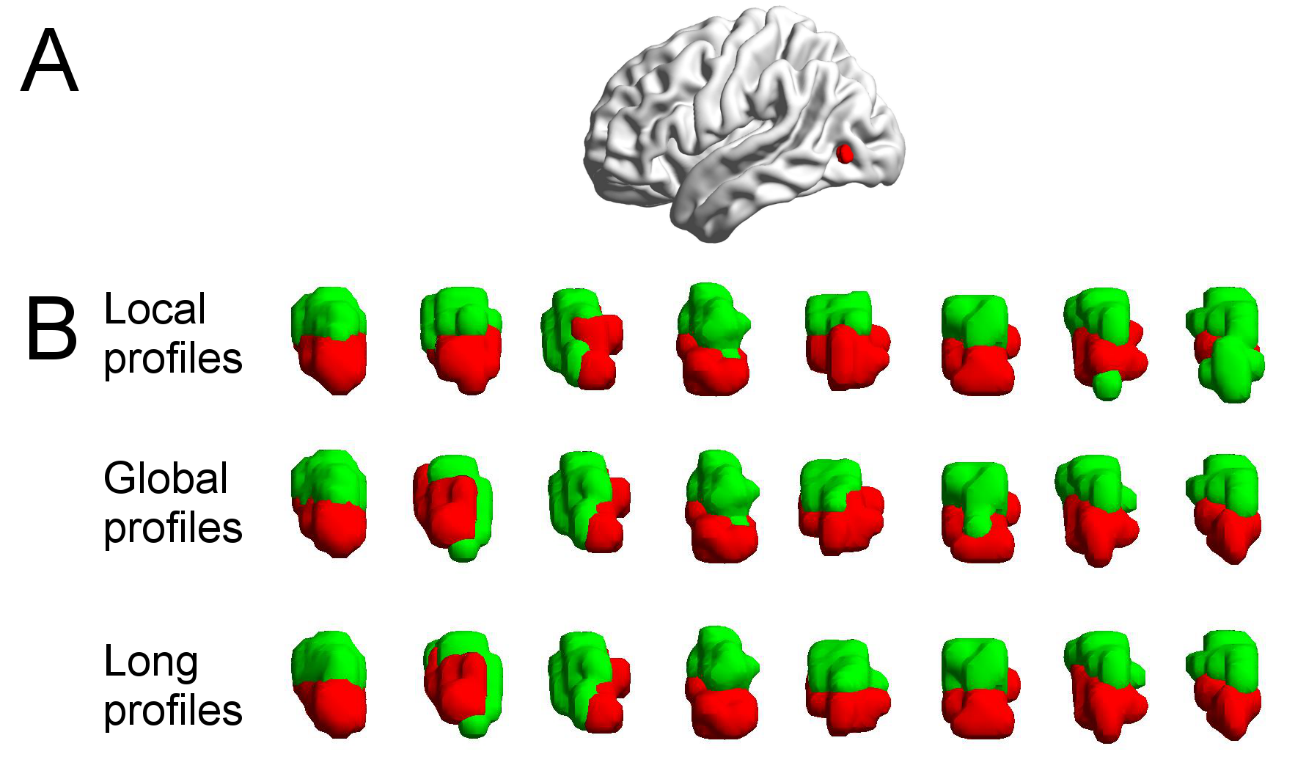


**FIGURE R1** The seed MT mask (A) and the parcellation results using the local, global, and long connectivity profiles (B). The first column in (B) showed the MPM results, and the others are individual example results.

1. **Reference**

Fischl, B., Van Der Kouwe, A., Destrieux, C., Halgren, E., Segonne, F., Salat, D.H., Busa, E., Seidman, L.J., Goldstein, J., Kennedy, D., Caviness, V., Makris, N., Rosen, B., and Dale, A.M. (2004). Automatically parcellating the human cerebral cortex. *Cereb Cortex* 14**,** 11-22. doi: 10.1093/cercor/bhg087.

Huk, A.C., Dougherty, R.F., and Heeger, D.J. (2002). Retinotopy and functional subdivision of human areas MT and MST. *J Neurosci* 22**,** 7195-7205. doi: 20026661.

Malikovic, A., Amunts, K., Schleicher, A., Mohlberg, H., Eickhoff, S.B., Wilms, M., Palomero-Gallagher, N., Armstrong, E., and Zilles, K. (2007). Cytoarchitectonic analysis of the human extrastriate cortex in the region of V5/MT+: a probabilistic, stereotaxic map of area hOc5. *Cereb Cortex* 17**,** 562-574. doi: 10.1093/cercor/bhj181.
